# Coherent anti‐Stokes Raman scattering (CARS) spectroscopy in Caenorhabditis elegans and Globodera pallida: evidence for an ivermectin‐activated decrease in lipid stores

**DOI:** 10.1002/ps.4707

**Published:** 2017-09-25

**Authors:** Justyna P Smus, Elizabeth Ludlow, Nicolas Dallière, Sarah Luedtke, Tual Monfort, Catherine Lilley, Peter Urwin, Robert J Walker, Vincent O'Connor, Lindy Holden‐Dye, Sumeet Mahajan

**Affiliations:** ^1^ Institute for Life Sciences and Department of Chemistry University of Southampton Southampton UK; ^2^ Biological Sciences University of Southampton Southampton UK; ^3^ Centre for Plant Sciences, School of Biology, Faculty of Biological Sciences University of Leeds Leeds UK

**Keywords:** potato cyst nematode, metabolism, Raman spectroscopy, abamectin, nematicide, starvation, Caenorhabditis elegans, Sudan Black, seed treatment

## Abstract

**BACKGROUND:**

Macrocyclic lactones are arguably the most successful chemical class with efficacy against parasitic nematodes. Here we investigated the effect of the macrocyclic lactone ivermectin on lipid homeostasis in the plant parasitic nematode Globodera pallida and provide new insight into its mode of action.

**RESULTS:**

A non‐invasive, non‐destructive, label‐free and chemically selective technique called Coherent anti‐Stokes Raman scattering (CARS) spectroscopy was used to study lipid stores in G. pallida. We optimised the protocol using the free‐living nematode Caenorhabditis elegans and then used CARS to quantify lipid stores in the pre‐parasitic, non‐feeding J2 stage of G. pallida. This revealed a concentration of lipid stores in the posterior region of J2 s within 24 h of hatching which decreased to undetectable levels over the course of 28 days. We tested the effect of ivermectin on J2 viability and lipid stores. Within 24 h, ivermectin paralysed J2 s. Counterintuitively, over the same time‐course ivermectin increased the rate of depletion of J2 lipid, suggesting that in ivermectin‐treated J2 s there is a disconnection between the energy requirements for motility and metabolic rate. This decrease in lipid stores would be predicted to negatively impact on J2 infective potential.

**CONCLUSION:**

These data suggest that the benefit of macrocyclic lactones as seed treatments may be underpinned by a multilevel effect involving both neuromuscular inhibition and acceleration of lipid metabolism. © 2017 The Authors. *Pest Management Science* published by John Wiley & Sons Ltd on behalf of Society of Chemical Industry.

## 1 INTRODUCTION

Ivermectin and abamectin are two members of the class of macrocyclic lactone compounds known as avermectins.^1^ Abamectin is a mixture of avermectin B1a and avermectin B1b.^1^ This class of compounds are effective as antiparasitics^2^ and pesticides.^3^ Abamectin is increasingly being adopted as a seed treatment for crop protection against plant parasitic nematodes.^4^, ^5^, ^6^, ^7^

In this study, we have investigated the effect of ivermectin on the potato cyst nematode (PCN) *Globodera pallida*. This is one of two main species belonging to the PCNs that are reported to cause losses of up to 50% of total crop yield.^8^ Unhatched second stage juvenile *G. pallida* (J2) can survive in cysts for up to 30 years without the host plant.^9^ Once J2 s have hatched, the survival of this infective, non‐feeding, pre‐parasitic stage depends upon neutral lipid reserves, depletion of which has been associated with reduced motility and infectivity.^10^

We have used Coherent anti‐Stokes Raman scattering (CARS) spectroscopy, which is a label‐free imaging technique, to quantify the effect of ivermectin on lipid stores in *G. pallida*. CARS is a non‐invasive and non‐destructive optical technique that can provide rapid selective mapping of biochemicals. Lipids may be selectively imaged using their characteristic vibration corresponding to the large number of methylene groups in alkyl chains of lipids. Previously, CARS has been used as a tool for metabolic profiling of *Caenorhabditis elegans* and to analyse lipid distribution in different developmental stages and in mutants defective in feeding or metabolism.^11^, ^12^

Here, we report the first use of CARS spectroscopy in a plant parasitic nematode using *C. elegans* as a benchmark for optimisation of the technique. We describe the regional distribution of lipid stores in *G. pallida* and show that ivermectin triggers a significant acceleration in the depletion of lipid reserves post‐hatching. We discuss this in the context of the use of macrocyclic lactones as seed treatments for crop protection.

## 2 EXPERIMENTAL METHODS

### 2.1 Nematode maintenance and culture

*Caenorhabditis elegans* (N2 Bristol strain) nematodes were maintained at room temperature on nematode growth medium, NGM 13agar plates seeded with *Escherichia coli* OP50 as a food source, according to standard protocols.^13^ *Globodera pallida* cysts were harvested from infected potato plants as previously described.^14^ To induce hatching, dried cysts were placed in a solution of 1 part potato root exudate to 3 parts double‐distilled (dd) H_2_O. (Full‐strength potato root exudate was obtained by soaking 80 g of washed potato root for 1 h in 1 L of distilled water.^15^) Significant numbers of J2 s typically began hatching 1 week after rehydration in the presence of potato root exudate. J2 s were used within 24 h of hatching. Prior to the experiments, the J2 s were washed in ddH_2_O to remove potato root diffusate.

### 2.2 Caenorhabditis elegans food deprivation for CARS analysis

To obtain age‐synchronised 1‐day‐old *C. elegans*, L4 larvae were picked onto seeded NGM plates and left to develop for 16 to 18h. The next day, the 1‐day‐old adults were either placed on an OP50 lawn (well fed) or subjected to food deprivation by being maintained for the indicated time on an agar plate without OP50. Well‐fed worms were compared with worms that had been food deprived for 0.5, 1, 1.5, 2, 4 and 24 h. For each time‐point, a plate containing around 45 worms was washed with 2 x 600 µL of M9 solution^13^ and transferred to an Eppendorf tube. Three wash cycles with 500 µL of M9 were performed and the worms were pipetted onto an NGM plate either seeded with *E. coli* (well fed) or without *E.coli* (starved).

Worms from each condition were washed with 2 x 600 µL M9 and transferred into an Eppendorf tube. Three further wash cycles with 500 µL of M9 were performed, as much supernatant as possible was discarded, and 150 µL of 4% formalin was added to the worm pellet after the final wash. These worms were fixed by incubation for 45 min at room temperature. Prior to imaging, three wash cycles with 500 µL of ddH_2_O were applied and the samples were kept at 4 °C until the mounting step.

### 2.3 Caenorhabditis elegans food deprivation and Sudan Black staining

*Caenorhabditis elegans* L4 worms were picked 32 h prior to staining to obtain a synchronised population of 1‐day‐old adults. Worms for the well‐fed control group were kept on food for the entire 32 h, whilst worms for the food‐deprived groups were transferred onto non‐seeded plates after 30 h (2 h food‐deprived), 27 h (5 h food‐deprived), 22 h (10 h food‐deprived) and 8 h (24 h food‐deprived). Worms of all groups were maintained at 20 °C. Every population of food‐deprived worms was paired with a control (fed) group on the same day. Ten worms were subject to analysis from each experimental group.

Worms were harvested from the plates using 500 µL of phosphate‐buffered saline (PBS) and transferred into a non‐stick 500‐µL Eppendorf tube. Two more wash cycles were performed before fixation with 1% paraformaldehyde (PFA v/v) for 15 min at room temperature.

These PFA‐fixed worms were subjected to three freeze–thaw cycles involving plunging the Eppendorf into liquid nitrogen and then a heat block, before worms were incubated for a further 5 min at room temperature. After a 10‐min incubation on ice, pelleted worms underwent three further wash cycles with 500 µL of PBS.

The worms were dehydrated by sequential exposure to increasing concentrations of ethanol (EtOH; 25%, 50% and 70%). Between each step, worms were incubated with rolling movements for 3 min in EtOH. As much EtOH as possible was then removed, and the worms were incubated overnight with 50% saturated Sudan Black (Fluka; Honeywell, Morris Plains, NJ, USA) in 70% EtOH. Sudan Black was prepared beforehand in 70% EtOH then filtered using a 0.22‐µm filter.

Stained worms were pelleted by a brief spin and rehydrated by three washing steps with a decreasing concentration of EtOH (70%, 50% and 25%). Two final wash cycles were then conducted with 1% PBS and stained worms were transferred onto a 2% agarose pad for observation.

### 2.4 Quantifying the effect of ivermectin on G. pallida motility; dispersal assay

Agar (2% w/v in water; High Gel Strength; Melford Laboratories, Ipswich, UK) was melted and cooled to 60 °C before supplementing with ivermectin or vehicle control at the indicated final concentration. A stock solution of 10 mM ivermectin was made in 100% EtOH from which dilutions were made to the required concentration with a final EtOH concentration of 0.05%. Ten millilitres of a sterile control or drug‐laced agar was dispensed into 55‐mm Petri dishes and allowed to set at 20 °C for 48 h before sealing the plates with parafilm and storing them at 20 °C until required. Plates were used within 1 month of being made. Prior to being used for assays, the plates were placed at room temperature and 100 µL of potato root diffusate (PRD) was spread evenly over their surface and allowed to dry for 10 min. *Globodera pallida* J2 larvae were hatched from cysts in a 1 in 4 dilution of PRD and collected up to 24 h post‐hatching. The J2 s were washed three times in distilled water before being used in the dispersal assay.

The plates were overlaid with a grid on which a central circle of 9 mm diameter provided a demarcation for the centre of each plate. A known number of J2 s (50 − 80) suspended in 5 µL of distilled water were placed at the centre of the test or control plates. The number of J2 s still residing within the central zone, or ‘origin’, was scored at 120 min and expressed as a percentage of the total number of worms on the plate. Worms lying across the boundary between the inner and outer zones were included in the score for the inner zone demarcated by the circle. The experiment was repeated six times on two different days.

### 2.5 Exposing G. pallida to ivermectin prior to CARS analysis

One day after hatching, *G. pallida* J2 s were incubated for 24 h in tubes containing 1 µm ivermectin or vehicle control.

### 2.6 Preparing specimens for CARS imaging

Nematode samples were lightly fixed in 4% formalin at room temperature for 45 min and then stored at 4 °C prior to analysis. Worms were mounted between two coverslips. A small square of folded parafilm was cut with scissors to produce a well and placed between the cover slips to form a spacer and avoid damage to the worms. A small droplet of the nematode sample containing the fixed worms was placed in the well.

### 2.7 CARS imaging methodology

A Chameleon Ultra (Coherent Inc., Santa Clara, CA, USA) titanium sapphire (Ti: Sa) 100‐fs pulsed laser with a repetition rate of 80 MHz was used. The fundamental pump beam at 835 nm was split into two beams, with one pumping an optical parametric oscillator (OPO) (Semi‐Automatic; APE GmbH, Berlin, Germany) to generate the Stokes beam at >1050 nm. Both the pump and the Stokes beam were stretched by passing through 10 cm of SF10 glass flats to improve the spectral resolution of imaging. For imaging of lipids, the CH_2_ stretching frequency at 2845 cm^−1^ for neutral lipids was targeted using the pump beam at 835 nm and the OPO tuned to 1097 nm. The two beams were temporally and spatially overlapped by using a delay stage (LTS203; Thorlabs, Newton, NJ, USA) and a dichroic mirror as a beam combiner. Galvanometer mirrors (GVSM002/M) were used for laser scanning the two collinear beams coupled into an inverted microscope (Ti‐U; Nikon, Tokyo, Japan) for imaging the specimen. CARS emission was optimised by alignment of the spatial and temporal overlay. Pixel dwell times were <30 µs. The blue‐shifted CARS signals from the specimens were read out in the Epi (back scattering) configuration. This minimised the CARS background. A Nikon 20x objective (0.75 NA) was used for imaging. The total power applied was <20 mW during imaging.

### 2.8 Image analysis

Images (10) were taken for each individual sample and at each time‐point. An area of 125 x 125 µm was scanned using scanimage (Janelia Farm, Ashburn, Virginia, USA) to generate highly resolved images at the optical diffraction limit with 1024 x 1024 pixels. For each time‐point, three worms were imaged. On each image, an area of interest was drawn and the number of pixels corresponding to the area was determined in imagej (US National Institutes of Health, Bethesda, MD, USA; http://imagej.nih.gov/ij/). Photomultiplier (PMT) shot noise occurring in the images was smoothed out using average filtering. Images were further processed by thresholding to the background from the specimen for consistency to quantify the lipid stores. imagej was used to calculate the area occupied by the lipid stores for each 2D image. The ratio between the number of pixels corresponding to the area occupied by lipid stores and the number of pixels of the distinct regions of interest was determined and used to compare different time‐points. Nematodes at the same life‐cycle stage were picked and hence were very similar in size and anatomy. The region of interest was selected such that it corresponded to the same number of segments and therefore was consistent between different worms.

The images shown in the figures are representative of 10 worms from two independent samples.

### 2.9 Statistical analysis

Data are expressed as mean ± standard error of the mean. Significance was determined using one‐way analysis of variance (ANOVA) with a Bonferroni post hoc test and a significance level of *P* < 0.05.

## 3 RESULTS

### 3.1 CARS imaging of lipid stores in C. elegans

**Figure 1:**
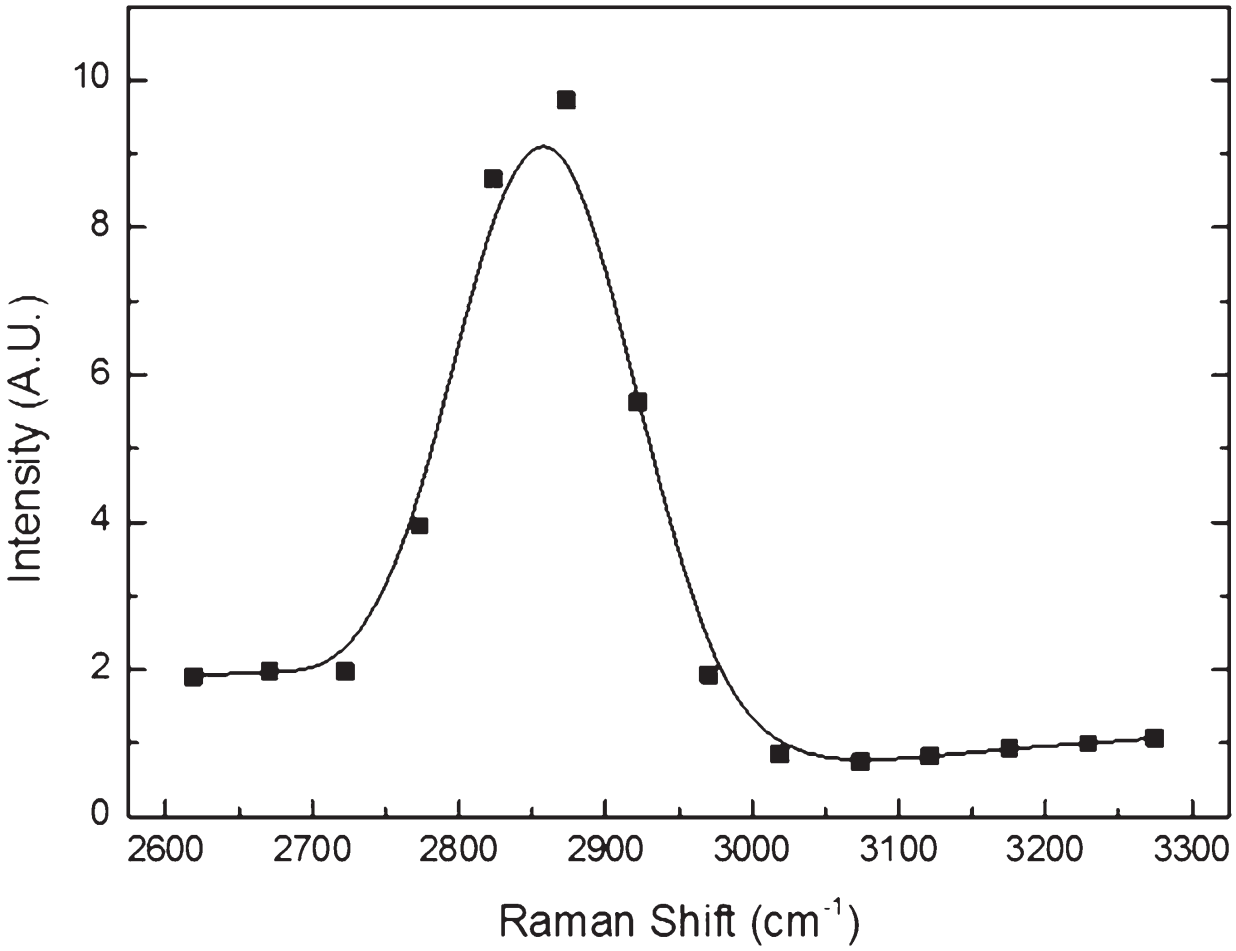
A Coherent anti‐Stokes Raman scattering (CARS) spectrum from Caenorhabditis elegans. This shows that the peak is at ∼2850 cm^‐1^. This corresponds well to the vibrational frequency expected for neutral lipids in the C‐H stretching region and provides validation of this approach for detection of lipid.

A CARS spectrum was acquired of lipid‐rich regions in *C. elegans*. It is presented in Fig. 1 and shows the characteristic CH_2_ stretching frequency in a typical dispersive peak. Within the limits of spectral resolution in these assays, estimated to be ∼30 cm^‐1^, it justifies the use of 2845 cm^‐1^ for mapping lipids in the specimens in this work.

**Figure 2:**
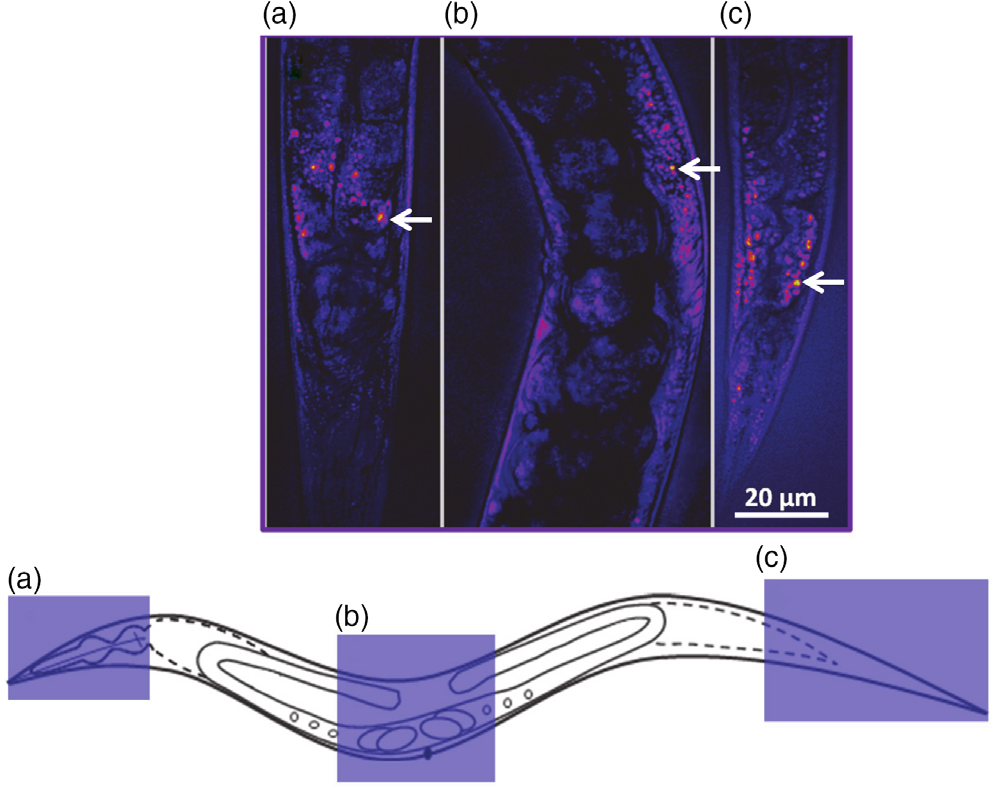
Representative CARS images of different regions, (a) head, (b) abdomen and (c) tail, in 1‐day‐old adult C. elegans. The corresponding segments are shown in the schematic below the images. CARS images were obtained by tuning to the ‐CH_2_ stretching frequency at 2845 cm^‐1^ for lipids. Lipid‐rich areas (lipid stores) appear as bright red/yellow puncta in the images, as indicated by the arrows. The image shown is representative of 10 similar images taken for each time‐point.

To evaluate the distribution of lipid droplets, images covering the head, abdomen (middle) and posterior/tail in adult (1‐day‐old) *C. elegans* worms were acquired (representative images of more than 10 similar images are shown in Fig. 2). We found that the highest lipid droplet concentrations were in the posterior/tail region, which correlates with the caudal region of the intestine. There was also a substantial amount of lipid present in the middle abdominal part of the body. In the upper part of the body, while the lipid signals were distributed, droplets were only visible around the pharynx.

### 3.2 Effect of starvation on lipid stores in C. elegans

**Figure 3:**
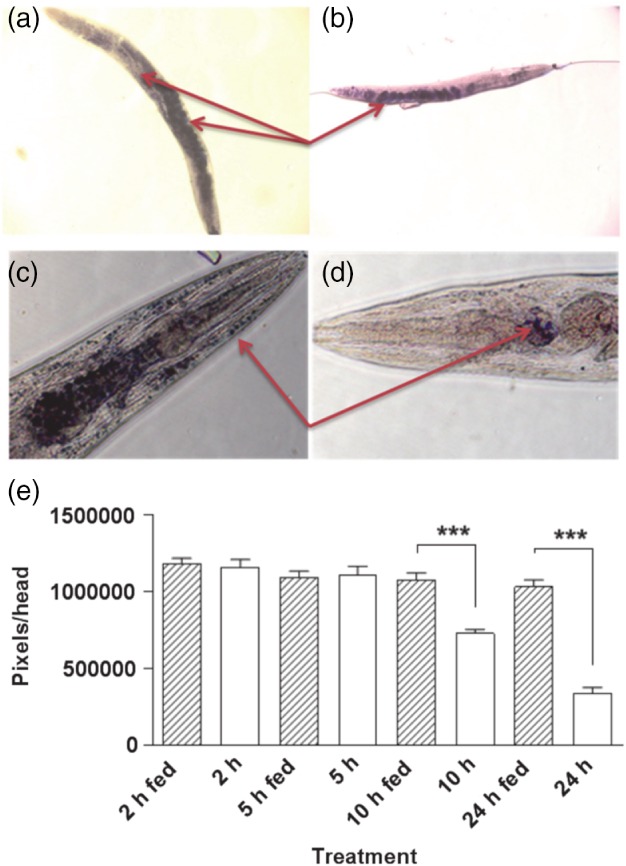
Sudan Black staining of wild‐type 1‐day‐old adult C. elegans worms which were either well‐fed or starved. (a − d) Photographs of well‐fed worms (a, c) and worms that had been food‐deprived for 24 h (b, d). Red arrows indicate the Sudan Black staining. (e) Sudan Black staining in C. elegans at different stages during food deprivation. The intensity was measured by highlighting the stained area as a region of interest. Pixels in a specified area were counted as the intensity. Every population of food‐deprived worms (open bars) was paired with a control group (hatched bars). During the first 5 h of food deprivation, there was no reduction in Sudan Black staining. After 10 h in the absence of food, there was a significant reduction in the level of Sudan Black staining and an even further decrease after 24 h of food withdrawal (n ≥ 10; mean ± standard error of the mean; ***P < 0.001).

**Figure 4:**
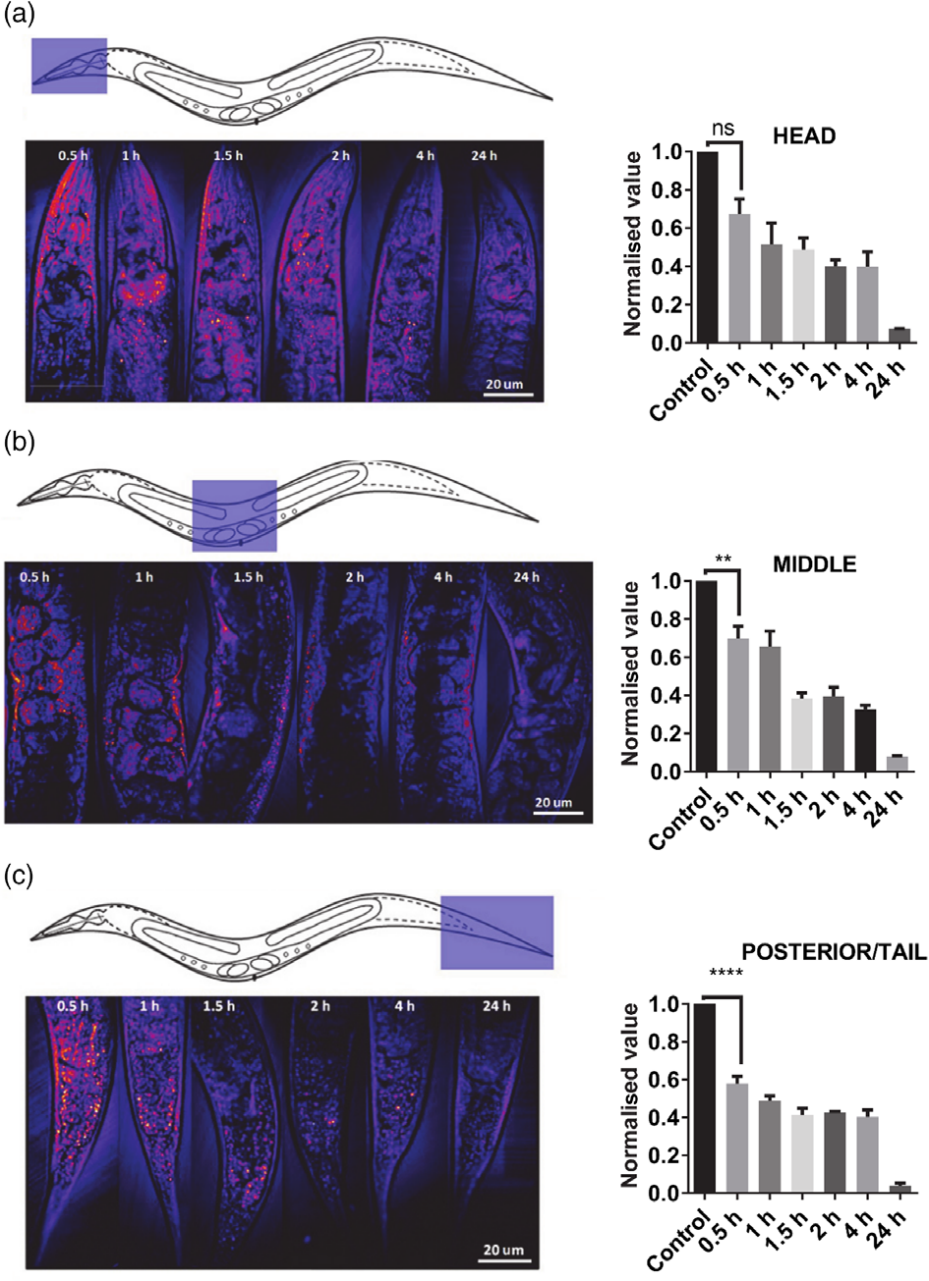
CARS images of regions of interest (a, head; b, middle; c, posterior/tail) from C. elegans after different times without food. The area of the worm from which images were collected is indicated in the left panels accompanied by representative images of the CARS signal (bright puncta correspond to the lipid vibrational frequency). Images from left to right were collected at increasing periods of food deprivation (0.5 to 24 h) and over this time‐course the abundance of the bright signal, i.e. the lipid vibrational frequency, decreases. Values have been normalized to those of control samples (well‐fed worms) and averaged from two replicates, each with n = 3. ****P < 0.0001; ***P < 0.001; **P < 0.01, and *P < 0.05).

We compared the ability of CARS to detect depletion of lipid stores with that of a standard histological approach using Sudan Black. This fixative‐based method was selected for the comparison as it has been shown to be more reliable for quantifying lipids than feeding worms vital dyes.^16^ One‐day‐old *C. elegans* hermaphrodites were subjected to starvation. Sudan Black staining was not changed in the first 5 h of food deprivation, whilst a significant depletion was observed at 10 h and an even greater loss after 24 h (Fig. 3). In marked contrast, CARS tuned to the vibrational frequency of lipid detected a dramatic decrease in signal within 30 min of food deprivation in the middle and posterior regions and more than 50% reduction in signal within 1.5 h of starvation in all three regions assayed (Fig. 4). After 24 h there was almost no CARS signal detectable. This suggests that, contrary to observations made with Sudan Black or Nile Red, food deprivation in *C. elegans* triggers an immediate increase in lipid metabolism and depletion in lipid stores. We conclude that CARS is a more sensitive approach than histological staining for the detection of changes in lipid content. Our data indicate that CARS is better suited to the detection of the kinetics of neutral lipid depletion, which is particularly salient for resolution of changes in response to environmental or chemical perturbants.

### 3.3 Imaging lipid stores by CARS in G. pallida J2s

**Figure 5:**
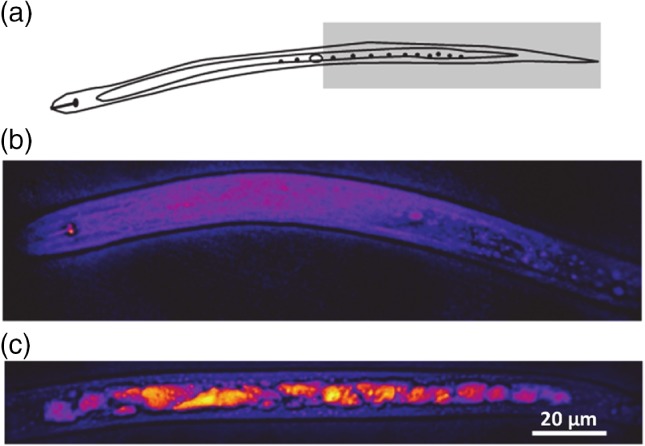
CARS images of G. pallida J2s. (a) The regions of the worm that were imaged; unshaded is the anterior/head region and shaded is the posterior/tail region. (b) The CARS signal corresponding to the lipid vibrational frequency from the anterior/head region is very weak. (c) In contrast, the CARS signal from the posterior/tail region is strong, as indicated by the extensive bright yellow/red regions.

Lipid stores are implicated in the infective potential of PPN.^10^ These investigations have thus far relied on biochemical and histological approaches such as Sudan Black staining, as described above. To evaluate whether CARS provides improved resolution, we investigated the distribution of lipid droplet images over the whole *G. pallida* J2 (Fig. 5). The nematode was imaged in two parts, as indicated in Fig. 5a: upper (head − abdomen; Fig. 5b) and lower (abdomen − tail; Fig. 5c) regions of the body. There was a very weak signal from the anterior region around the oesophageal, pharyngeal region apart from a bright, punctate signal from the stylet knob, which is the posterior‐most tip of the stylet organ and is used as a lance‐like structure to facilitate hatching, root invasion and feeding (Fig. 5b). As for *C. elegans*, the CARS signal tuned to the vibrational frequency of neutral lipid was strongest from the posterior region of the body of *G. pallida* (Fig. 5c). This region corresponds to the terminal end of the intestine. Similar to *C. elegans*, there was also a strong CARS signal from the middle abdominal part of the body. Again, this is similar to the results with *C. elegans* and is consistent with the intestinal cells being a major site for lipid storage.

### 3.4 Following lipid content post‐hatching in G. pallida J2s

**Figure 6:**
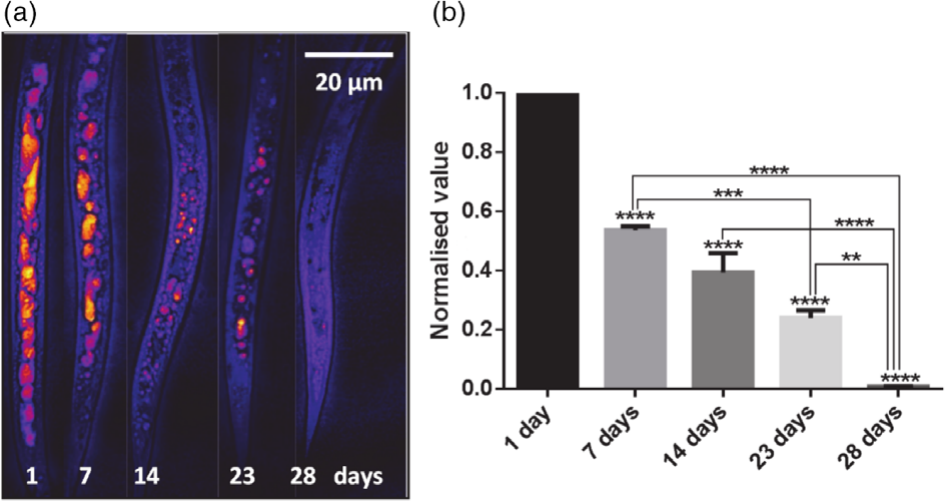
(a) Representative CARS images of the posterior/tail region of Globodera pallida J2s after hatching. The number of days after hatching is indicated on each image. The CARS signal corresponding to the lipid vibrational frequency (bright yellow/red areas) is depleted over time post‐hatching. (b) Quantitative analysis of lipid stores from CARS images showed a decrease after hatching in J2 G. pallida. Values have been normalized to values corresponding to 1‐day‐old worms and averaged from two independent data sets, each with n = 10. ****P < 0.0001; ***P < 0.001; **P < 0.01, and *P < 0.05.

After hatching from cysts, the infectivity of *G. pallida* is related to the amount of lipid stores,^10^ and therefore understanding the depletion rate of lipids has the potential to be informative for nematicidal treatment and crop protection. Representative CARS images of lipid stores in *G. pallida* recorded on different days after hatching are shown in Fig. 6a. The CARS analysis indicates that the lipid stores are gradually depleted over a time‐course of days. This contrasts with the much faster rate of depletion observed in starved *C. elegans* which are far more motile than *G. pallida* J2s. A quantification of the data showed that nearly 50% depletion occurred over the first 7 days and that there was almost a linear depletion of ∼10% per week (Fig. 6b). This observation is consistent with the plant parasitic nematode *G. pallida* having a lower overall metabolic rate than the free‐living *C. elegans*.

### 3.5 Effect of ivermectin on G. pallida viability and lipid stores

**Figure 7:**
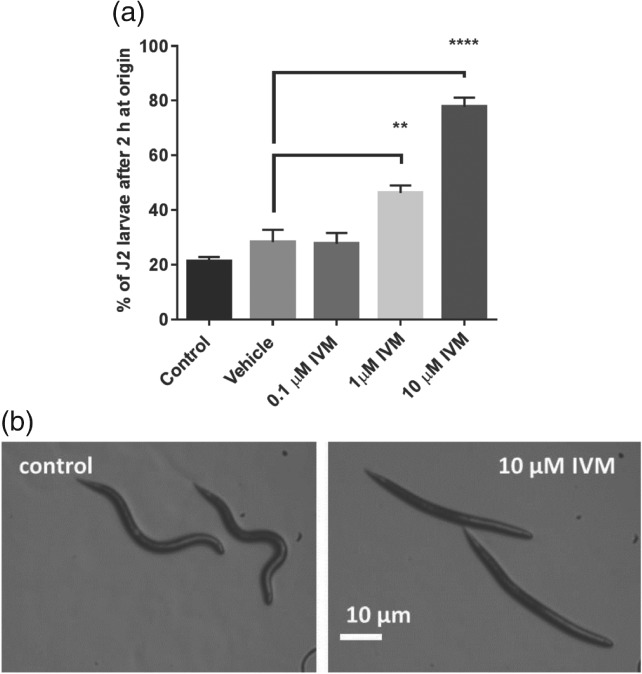
Ivermectin inhibits the motility of G. pallida. (a) Ivermectin inhibited dispersal of J2s from the centre, ‘origin’, of an agar plate in a concentration‐dependent manner after 2 h of exposure. Data are mean ± standard error of the mean. The experiment was repeated six times on two different days. Vehicle for the ivermectin was 0.5% ethanol, and 0.5% ethanol was incorporated in the control plate. (b) Two hours of exposure to 10 µM ivermectin causes a marked flaccid paralysis, characterised by the loss of postural shape of the worms. Each image is representative of 10 samples.

**Figure 8:**
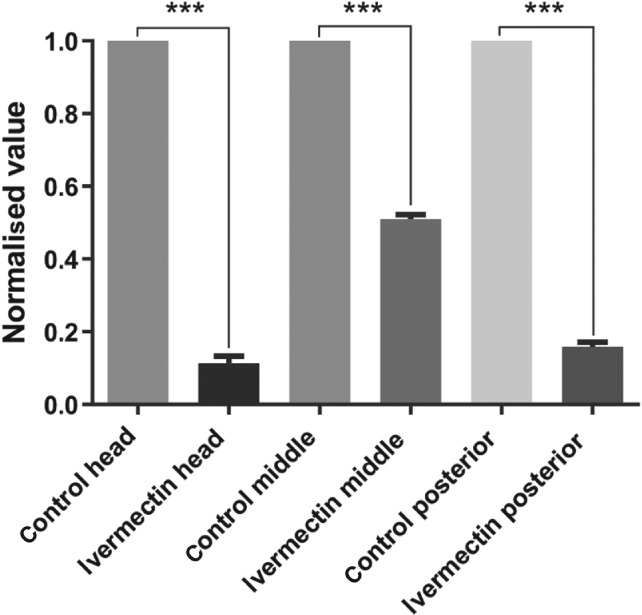
CARS analysis of J2 G. pallida incubated with ivermectin. J2s were selected within 1 day of hatching and incubated with 1 µM ivermectin or vehicle (control) for 24 h. CARS signals corresponding to the lipid vibrational frequency were measured from the head, middle and posterior region of each worm. The signals from the ivermectin‐treated worms were normalised with respect to the vehicle control. n = 3. ***P < 0.001.

In the dispersal assay, which provides a read‐out of the effect of ivermectin on J2 motility, ivermectin elicited a concentration‐dependent inhibition of dispersal with a rapid onset of action occurring within 2 h (Fig. 7a). J2s exposed to 10 µM ivermectin exhibited a flaccid paralysis (not turgid when prodded) (Fig. 7b), consistent with its mode of action involving increased glutamatergic inhibitory signalling.^17^ In this same paradigm, J2s were exposed to a submaximal concentration of 1 µM ivermectin for 24 h and subjected to CARS analysis. This revealed an acceleration of lipid loss in the ivermectin‐treated J2s compared with vehicle controls (Fig. 8).

## 4 DISCUSSION

The macrocyclic lactones are arguably one of the most successful chemical classes with selective toxicity against nematodes relative to mammals.^18^ The mode of action of ivermectin has been extensively investigated in *C. elegans* 17, 19 and in animal parasitic nematodes.^20^, ^21^, ^22^ Together, these studies have resolved a family of glutamate‐gated chloride channels (GluCls) as a major target of the macrocyclic lactones in animal parasitic nematodes of the gastrointestinal tract.^17^, ^21^, ^22^ The macrocyclic lactones have been shown to irreversibly activate GluCls,^17^, ^19^ bringing about neuromuscular inhibition^23^ and paralysis, underpinning their anthelmintic action. In the plant parasitic nematode *Meloidogyne incognita*, it has been shown that treatment of pre‐infective J2s with either abamectin or avermectin (avermectin B_2a_‐23‐one) inhibits their motility and limits their ability to invade roots.^5^, ^24^ The ability of the chloride channel antagonist picrotoxin to block the inhibitory effect of avermectin on *M. incognita* motility^24^ may implicate a chloride channel in the bioactivity of macrocyclic lactones in these plant parasitic species of nematode. Whether or not this is a GluCl, similar to the target in animal parasitic nematodes, remains to be determined. Intriguingly, there is growing evidence that there may be additional targets for the macrocyclic lactones over and above their potent effects mediated through GluCls.^25^ For example, recently it has been reported that the macrocyclic lactone abamectin may exert some of its effects through nicotinic acetylcholine receptors.^26^ Further analysis of the effect of ivermectin against the animal parasitic filarial nematode *Brugia malayi* 27 using transcriptomics has suggested an effect on metabolism involving oxidative phosphorylation.^28^

In this study, we have focused on the effect of ivermectin on lipid metabolism. Lipid stores are essential to the viability and fecundity of nematodes. Lipid homeostasis has previously been investigated in detail in *C. elegans*, providing evidence for neuronal regulation of energy homeostasis.^29^, ^30^ Whilst the role of replete lipid stores in the infective potential of plant parasitic nematodes is well established, less is known about energy homeostasis in these pests. Moreover, the impact of chemical pest control agents on lipid stores in PPNs remains under‐explored and could provide further insight into mechanisms of action.

To facilitate this investigation, we have optimised CARS for a kinetic analysis of lipid depletion using *C. elegans*. Previously, CARS analysis confirmed that the major lipid stores in adult hermaphrodite *C. elegans* are in the hypodermis and intestine.^16^ In our study, the distribution of the CARS signal in adult *C. elegans* was similar to that previously reported by Hellerer *et al*.^11^ It confirmed the localisation of a signal in the intestine and also a strong signal running along the inner face of the worm cuticle, a region corresponding to the hypodermis, and thus supporting the interpretation that this tissue is a site of lipid storage. Previous studies have tracked lipid depletion in *C. elegans* during starvation using conventional histological staining methods.^11^, ^29^, ^30^, ^31^ For example, it was shown that there was no change in Nile Red staining of *C. elegans* after 3 h of starvation, with a decrease occurring between 3 and 18 h.^30^ Similarly, using Oil‐Red‐O, it has been shown that 4 to 6 h of food deprivation can deplete the lipid signal.^32^ We replicated this using Sudan Black and observed a similar time‐course for depletion as in these previous studies.^30^ Together, the findings of these studies using histological stains as markers for lipid suggest that at early time‐points of starvation, i.e. within the first few hours, lipid stores are not significantly depleted. However, these histological methods may differ from CARS in that they may also label phospholipids, as demonstrated for Nile Red.^33^ Furthermore, there is a report that Nile Red and BODIPY label acidified intracellular compartments of *C. elegans* that are not related to the major fat stores.^34^ By using CARS in *C. elegans* subjected to food deprivation, we have detected a previously unresolved decrease in lipid content that occurs within 30 min of the onset of food deprivation. This suggests that lipid stores are subject to short‐term dynamic regulation and are under direct neuroendocrine control in addition to previously reported transcriptional responses.^31^ Importantly, it exemplifies the power of the CARS approach to resolve discrete aspects of energy homeostasis in microscopic nematodes that cannot be detected by histological methods.

The value of this for probing the biology of parasitic nematodes is highlighted by our use of CARS as a method for monitoring the distribution and dynamics of lipid distribution in the plant parasitic nematode *G. pallida*. We have extended previous information on the lipid composition of *G. pallida* J2s obtained with biochemical and labelling techniques^35^, ^36^ to a detailed description of the distribution and dynamics of lipid stores in this economically important pest. We analysed the J2 life stage at different time‐points post‐hatching. Interestingly, given that the pre‐infective J2s are non‐feeding and must survive on their energy reserves until they establish a feeding site in their host plant, the CARS signal from these nematodes was much stronger than from *C. elegans*. This provides evidence that J2s hatch from their egg replete with lipid reserves to facilitate survival during the post‐hatch host‐finding period. The lipid appears to be highly concentrated in the posterior/tail region of the worm in a distribution consistent with lipid storage in the gut. However, there was little evidence for storage in hypodermal tissue as was observed for *C. elegans*.

The depletion of lipid in *G. pallida* J2s occurs slowly post‐hatching, with a 50% reduction after 7 days compared with 1.5 h in adult *C. elegans*. This is in line with the recognition of J2 as a relatively metabolic inactive life‐stage, somewhat similar to the *C. elegans* dauer stage.^37^ 28 days post‐hatching, the lipid stores in *G. pallida* would appear to be completely depleted.

We have shown that ivermectin brings about a rapid paralysis of *G. pallida*, similar to its effect on *Meloidogyne incognita* 24 and consistent with an inhibitory action on nematode neuromuscular function. In addition, we observed that lipid stores were more rapidly depleted in ivermectin‐treated worms compared with untreated worms. This observation was made after 24 h of treatment with ivermectin, i.e. at a time‐point at which the worms were rendered immobile by the ivermectin treatment. This result is rather counterintuitive as it might be expected that the inactivity of the nematodes would place less demand on their lipid reserves. In this context, it is interesting to note that ivermectin and other macrocyclic lactone analogues have been shown to reduce lipid accumulation in mice^38^ and there is interest in the impact of these compounds on metabolic activity.

Our data are consistent with the view that the mode of action of macrocyclic lactones used in seed treatment^4^ is a multi‐level effect, involving inhibition of neuromuscular function and acceleration of lipid metabolism, which both have potential to decrease infectivity. In support of this interpretation, it has been shown that treatment of *G. rostochiensis* with low doses of the carbamate nematicide oxamyl led to an increase in lipid content and the worms subjected to this treatment showed a correspondingly enhanced infectivity in a root invasion assay.^36^ Furthermore, these observations of an effect of ivermectin on energy homeostasis in *G. pallida* resonate with an increasing realisation that the mode of action of ivermectin across a range of invertebrate organisms involves targets other than the GluCls^25^ and that these modes of action may underpin effects of ivermectin in mammalian tissues.

## 5 CONCLUSION

The macrocyclic lactone compound ivermectin exerts a lipid‐depleting effect on *G. pallida*. This suggests that this chemical class, which includes abamectin, a seed treatment used as a crop protectant, may compromise energy homeostasis in the plant parasitic nematode to bestow crop protection.
